# Urodynamic outcome of parasacral transcutaneous electrical neural stimulation for overactive bladder in children

**DOI:** 10.1590/S1677-5538.IBJU.2014.0303

**Published:** 2015

**Authors:** Ubirajara Barroso, Marcelo Tomás Carvalho, Maria Luisa Veiga, Marília Magalhães Moraes, Carolina Coelho Cunha, Patrícia Lordêlo

**Affiliations:** 1 CEDIMI (Centro de Distúrbios da Micção em crianças), Departamento de Urologia da Divisão de Urologia Pediátrica, Escola Bahiana de Medicina e Universidade Federal da Bahia Salvador, Bahia; 2 Universidade Federal da Bahia Salvador, Bahia

**Keywords:** Urodynamics, Transcutaneous Electric Nerve Stimulation, Child, Bladder

## Abstract

**Objective:**

To evaluate the urodynamic changes immediately after the first session (acute effect) and after the last session of parasacral TENS in children with idiopathic OAB.

**Materials and methods:**

We performed urodynamic evaluation immediately before and after the first session of parasacral TENS and immediately after the last session (7 weeks later). Only children with idiopathic isolated OAB were included. Patients with dysfunctional voiding were not included.

**Results:**

18 children (4 boys and 14 girls, mean age of 8.7) were included in the first analysis (urodynamic study before and immediately after the first session) and 12 agreed to undergo the third urodynamic study. Urodynamic before and immediately after the first session: There was no change in the urodynamic parameters, namely low MCC, low bladder compliance, presence of IDC, the average number of IDC, or in the maximum detrusor pressure after the first exam. Urodynamic after the last session: The bladder capacity improved in most patients with low capacity (58% vs. 8%). Detrusor overactivity was observed in 11 (92%) before treatment and 8 (76%) after. There was not a significant reduction in the average number of inhibited contractions after TENS (p=0.560) or in the detrusor pressure during the inhibited contraction (p=0.205).

**Conclusion:**

There was no change in the urodynamic parameters immediately after the first session of stimulation. After the last session, the only urodynamic finding that showed improvement was bladder capacity.

## INTRODUCTION

TENS has been used in the treatment of pediatric OAB bladder (1–3). Two randomized clinical trials have shown that parasacral TENS is an effective treatment of OAB in children (4, 5). Some authors have reported improvement in the urodynamic parameters during or after posterior tibial electrical neural stimulation (PTNS) in adults (4–6). However, despite that the effectiveness of TENS in children with idiopathic OAB has been demonstrated subjectively, to our knowledge, the urodynamic outcome after treatment has not been reported. The aim of this study is to evaluate the urodynamic changes immediately after the first session (acute effect) and after the last session of parasacral TENS in children with idiopathic OAB.

## MATERIALS AND METHODS

We performed urodynamic evaluation immediately before and after the first session of parasacral TENS and immediately after the last session (7 weeks later). The inclusion criteria was: 1 children with isolated OAB, defined as the presence of urgency, with or without daytime incontinence, associated with a bell shaped/tower uroflow curve and post void residual of less than 10% of the expected bladder capacity and less than 20 mL; and 2 patients without any neurological problem or any anatomical disease of the lower urinary tract. The exclusion criteria included the diagnosis of dysfunctional voiding or any anatomical or neurological problem of the lower urinary tract during the treatment, or the impossibility to perform the urodynamic study. This study was approved by our institutional Ethical Committee and all parents signed an informed consent. According to our IRB we were allowed to use urodynamic for any children with symptoms of OAB that underwent TENS.

Patients underwent 20 sessions of parasacral TENS with a duration of 20 minutes each. The frequency of current used was 10 HZ, the pulse width 700µ seconds and the current intensity was at the sensitivity threshold. Pads were placed on each side of S3. Three urodynamic studies (Dy-named®, São Paulo, Brazil) were performed: immediately before and after the first session and immediately after the last TENS session. For this procedure, two catheters 4 and 6 Fr were introduced in the bladder and one in the rectum to record the abdominal pressure. The rate of bladder feeling was 10% of the expected bladder capacity (30 x age+30) per minute (7). The same professional (MTC) performed all exams. The bladder and abdominal catheters were in place during the first parasacral TENS.

The endpoints used to evaluate the urodynamic effect of the parasacral TENS were: low MCC (less than 60% of the expected bladder capacity), compliance (less than 10), the presence of detrusor overactivity, the number of IDC, and the highest (maximum) pressure during IDC. For symptom evaluation we used visual analogic scale, where 0 was no improvement and 10 complete resolution of the symptoms.

We used SPSS 15.0 to perform the statistical analysis. Continuous variables were tested by the Wilcoxon test. A p value of less than 0.05 was considered significant.

## RESULTS

Eighteen patients were included in the first analysis (urodynamic study before and immediately after the first session) and 12 agreed to undergo the third urodynamic study and could be included in the third analysis. Of these, all patients had urgency before treatment, 9 had daytime incontinence and 4 had frequency. Ten patients had a history of UTI before the beginning of the treatment.

Urodynamic before and immediately after the first session (Table-1): 18 children (4 boys and 14 girls, mean age of 8.7, ±2.9) were evaluated. The urodynamic findings of the two studies were as follows: No patient had a low bladder compliance before treatment. All patients with low MCC maintained this finding after TENS. Of 16 patients with detrusor overactivity, one presented a stable bladder after the session. There was no change in the average number of IDC (p=0.84), nor in the maximum detrusor pressure (p=0.2) during the inhibited contraction before and after TENS.

**Table 1:** Urodynamic outcome after the first parasacral TENS (N=18).

	Before	After
Low CMC	12	12
Low compliance	4	3
Presence of IDC	16	15
Number of IDC (SD)€	6.5 (6.4)	6.3 (5.7)*
Higher IDC (SD)£	49.6 (35.1)	42.9 (38.7)*

€ Average

£ Average of the highest pressure during IDC

* p values were non-significant

Urodynamic after the last session (Table-2)-Twelve patients underwent the third urodynamic study and participated on this analysis. The bladder capacity improved in 6 patients. Detrusor overactivity was demonstrated in 11 (92%) before treatment and in 8 (73%) after. There was no significant reduction neither in the average number of inhibited contractions after TENS (p=0.560) nor in the detrusor pressure during the involuntary bladder contraction (p=0.205). One patient who had a stable bladder before treatment maintained this state after the procedure. Among 10 patients who had information available about the symptoms, 9 had complete resolution of the LUTS. Seven patients who had the symptoms improved after TENS still had detrusor overactivity.

**Table 2:** Urodynamic outcome before the treatment after the last parasacral TENS (N=12).

	Before	After
Low CMC	7 (58%)	1 (8%)
Presence of IDC	11 (92%)	8 (73%)
Number of IDC (SD)€	6.0 (6.9)	5.33 (5.5)*
Higher IDC (SD)£	49.7 (35.1)	42.9 (38.7)*

€ Average

£ Average of the highest pressure during IDC

* p values were non-significant

## DISCUSSION

We demonstrated in this study of children with isolated OAB that, after the end of treatment, parasacral TENS improved the symptoms, but the only improved urodynamic finding was the MCC. Kabay et al. found improvement in the urodynamic findings after 12 weeks of PTNS in adults patients with neurogenic detrusor overactivity secondary to multiple sclerosis (8). The average bladder volume during the first involuntary detrusor contraction on the first cystometry was 124.2 mL, while it was 217.5 mL after PTNS. MCC on standard cystometry was 199.7 mL, while it was 266.8 mL after stimulation. This last finding is in accordance with our results.

In our study, despite improving the symptoms, 73% of the patients still presented IDC and there was no change in the number of the IDC as well as in the pressure of the IDC before and after parasacral TENS. This confirms the findings of one previous study that demonstrated that the symptomatology can improve and the patients may persist with detrusor overactivity (9). We hypothesize that even after being treated, patients develop neuroplasticity but do not develop the capacity to inhibit the micturition reflex during the urodynamic study. However, this is not the case in the physiological bladder filling. Another interpretation is that the ENS modulates the symptom interpretation by the brain and, hence, there is no brain reaction to an IDC. Another hypothesis is that the urodynamic study is not reliable as a diagnosis method of OAB in children. The same explanations can be used to justify the absence of an acute urodynamic effect of TENS.

Amarenco et al. were the first to report data concerning acute stimulation and immediate cystometry modifications after PTNS (10). A total of 44 consecutive patients with urge incontinence, and frequency and urgency secondary to overactive bladder (37 with neurogenic and 7 with idiopathic OAB) were studied. Cystometry was done before PTNS and then it was repeated during the stimulation. PTNS was associated to significant improvement in IDC volume and to significant improvement in MCC. Kabay et al. evaluated the acute effects of PTNS on the urodynamic findings in adults with Parkinson disease and multiple sclerosis with neurogenic detrusor overactivity (8, 11). There was improvement in the average bladder capacity during the first involuntary contraction and in the MCC average before and during PTNS.

All three studies evaluated the urodynamic changes during the first session of ENS and differed from ours in that we evaluated it immediately after the first session. They found improvement in the urodynamic parameters and we did not. This difference could relate to the different timing of the urodynamic study. The urodynamic effect could be present during the session due to a pudendal nerve or interneurons stimulation and be suppressed after the end of the session. Furthermore, the difference could also be the result of different types of stimulation and the differences in the samples studied. However, even though some effect on the bladder function may exist during stimulation, our study shows that this effect does not last after the first session.

Regarding the limitations of this study, we did not evaluate the lower urinary tract function during the stimulation. Therefore, we cannot know if there is an effect on the bladder filling phase when the patient is being neurostimulated. Also, the number of patients who underwent the third urodynamic is small. However, urodynamic is an uncomfortable method for toilet-trained children and it is not part of the mandatory work-up for children with OAB. Also, because of the retrospective nature of the symptoms evaluation, we did not have available the visual analogic scale evaluation of some patients. Another limitation is that we did not compare the averages of bladder capacity, compliance and pressure during bladder contraction. Instead, we used these data as categorical variables. Despite having low power for statistical evaluation, using urodynamic findings in categorical variables leads us to better clinical interpretation, since we know how many patients normalized their urodynamic parameters.

We reported the urodynamic outcome of patients with isolated OAB treated by a specific type of ENS. Therefore, these results may not be extrapolated to children with OAB associated with dysfunctional voiding or for those who underwent other kinds of ENS.

## CONCLUSIONS

There is no change in the urodynamic parameters immediately after the first session of stimulation, demonstrating that there is no acute urodynamic effect of the parasacral TENS. Despite the symptoms of most patients have improved, after the last session, the only urodynamic finding that showed improvement was bladder capacity.

## ABBREVIATIONS

OAB =: Overactive bladder
ENS =: Electric neural stimulation
IDC =: Involuntary detrusor contraction
LUTS =: Lower urinary tract symptoms
MCC =: Maximum cystometric capacity
PTNS =: Posterior tibial electrical neural stimulation
TENS =: Transcutaneous electrical stimulation
